# A GPU Simulation Tool for Training and Optimisation in 2D Digital X-Ray Imaging

**DOI:** 10.1371/journal.pone.0141497

**Published:** 2015-11-06

**Authors:** Elena Gallio, Osvaldo Rampado, Elena Gianaria, Silvio Diego Bianchi, Roberto Ropolo

**Affiliations:** 1 S.C. Fisica Sanitaria, A.O.U. Città della Salute e della Scienza, Turin, Italy; 2 Department of Computer Science, University of Turin,Turin, Italy; 3 Department of Surgical Sciences, University of Turin,Turin, Italy; The University of Chicago, UNITED STATES

## Abstract

Conventional radiology is performed by means of digital detectors, with various types of technology and different performance in terms of efficiency and image quality. Following the arrival of a new digital detector in a radiology department, all the staff involved should adapt the procedure parameters to the properties of the detector, in order to achieve an optimal result in terms of correct diagnostic information and minimum radiation risks for the patient. The aim of this study was to develop and validate a software capable of simulating a digital X-ray imaging system, using graphics processing unit computing. All radiological image components were implemented in this application: an X-ray tube with primary beam, a virtual patient, noise, scatter radiation, a grid and a digital detector. Three different digital detectors (two digital radiography and a computed radiography systems) were implemented. In order to validate the software, we carried out a quantitative comparison of geometrical and anthropomorphic phantom simulated images with those acquired. In terms of average pixel values, the maximum differences were below 15%, while the noise values were in agreement with a maximum difference of 20%. The relative trends of contrast to noise ratio versus beam energy and intensity were well simulated. Total calculation times were below 3 seconds for clinical images with pixel size of actual dimensions less than 0.2 mm. The application proved to be efficient and realistic. Short calculation times and the accuracy of the results obtained make this software a useful tool for training operators and dose optimisation studies.

## Introduction

In recent years, several types of simulation software for radiological applications have been developed [1–7], considering the interaction between radiation, biological tissues and detector systems. These tools were implemented with the aim of optimising exposure parameters or image quality. The interest for the research in this field has been favoured by the growth in computer calculation potentiality.

Radiological images simulation requires the implementation of various components: primary beam and its attenuation, scatter radiation, noise, a virtual patient and a digital detector. Although the first three ones are well simulated with Monte Carlo methods, these methods have long calculation times, making them impossible to use for practical and didactic real time interactivity with users. Alternatively, analytical methods can be used which are able to calculate much faster, but the models used may be more complex to account for the processes involved. Moreover, they do not generally consider all the radiation components.

The primary beam should be polychromatic and reflect different filtrations, angles and anode materials of various types of radiological equipment. Various approaches can be used for obtaining spectra such as the Birch and Marshall [8] or the Boone and Seibert methods [9] and they can also be obtained from in air fluence maps [10]. The actual finite size of the X-ray source, namely the focal spot dimensions, can be simulated by summing the beams generated by several single point sources adjacent to each other [1].

In additional to the primary radiation, there is also the scatter radiation that is usually simulated with Monte Carlo methods. Some experiences with alternative approaches were also studied. These included analytical models for the first order of scatter by considering the various phantom voxels as scatter sources [11] or from experimental scatter maps [7].

Two kinds of datasets are commonly used for the simulation of the patient: voxel phantoms or boundary representation (BREP) phantoms [12]. The voxel phantoms can be anthropomorphic phantoms or anonymized hospital patients obtained from CT or MRI images. The BREP phantoms are computational human models that contain exterior and interior anatomical features of a human body using boundary representation methods.

Several strategies have been adopted to simulate the recent different digital detectors and the relative components. Commonly, the simulated detector is modelled to be a 2D array of elements [1, 10], with the actual size of the pixel reduced to a single point. Bontempi *at alt*. [13] simulated the detector as an absorber in which every material and component interacts with photons. In their code, they considered the absorber as a box with an input surface, a thickness and an output surface.

Another important component of the image acquisition system is the scatter removal grid. To our knowledge, only one study in literature has focused on the contribution of antiscatter grid [5], which enables the user to specify the transmission percentages of the primary image and the scattered one.

A simulation tool for evaluating new dose reduction techniques, should implement a proper simulation of the image noise.There are three main noise contributions for digital X-ray images: electronic noise, quantum noise and fixed pattern noise [14]. The importance of these contributions can be evaluated through experimental measurements on different types of detectors, by analysing the standard deviation trends of the acquired images, for different levels of dose. The noise can be computed during the image generation or in a post-processing phase [6].

In this study, a digital X-ray imaging system simulation tool based on graphics processing unit (GPU) computing and CUDA (Compute Unified Device Architecture) architecture is presented. Originally designed for accelerating the production of computer graphics, the GPU has proved to be a versatile platform for running massively parallel computation, without jeopardising reliability and accuracy. There are many advantages in using graphic hardware for processing the type of datasets encountered in medical physics: high memory bandwidth, high computation throughput, support for floating-point arithmetic and the lowest cost per unit of computation [15]. Since 2000, an ever-increasing number of publications have focused on the use of GPU as co-processor. The introduction of CUDA by NVIDIA, currently the most popular GPU computing API, has greatly simplified the development of distributed computing applications for GPU which has facilitated GPGPU (General-Purpose Computing on Graphics Processing Units) programming. CUDA provides a set of extensions to standard programming languages, like C, that allows for the straightforward implementation of parallel algorithms on the GPU [16]. Therefore, GPU computing has become a useful research tool for a wide range of medical physics applications: image reconstruction, dose calculation, treatment plan optimisation and image processing [15]. The improvements in GPU performance and programming allows for the physically-realistic simulation of X-ray imaging in interactive time.

Primary beam and its attenuation, noise, virtual phantom, scatter radiation contribution and three different detectors were used for the application and a comparison of the computed images with those acquired was carried out in order to validate the software.

## Material and Methods

The interaction of the primary beam with a virtual phantom was modelled according to a forward projection method through voxel volumes, described in [17]. The punctual and polychromatic source was an ordinary X-ray tube with which the user is able to adjust the amount of energy and intensity. The different beams were obtained from IPEM (Institute of Physics and Engineering in Medicine) *Report 78-Spectrum Processor* program by setting traditional clinical X-ray tube features and dividing the spectrum into 5 keV energy bins. For every beam, we created an input file indicating photon fluence normalized at a distance of 1 m (Φ_i_) and energy (E_i_) for each energy bin. The virtual body consisted in a 3D voxel matrix (voxel size: 0.62 x 0.62 x 0.62 mm^3^) generated from CT images. The linear attenuation coefficient (μ) of each voxel was estimated from the HU value: μ_voxel_ = (HU/1000+1) ·μ_water_. For μ_water_ the value corresponding to 60 keV was considered. This was the effective energy corresponding to the 120 kVp X-ray beam of the anthropomorphic phantom CT. For the other energies μ_voxel_ was multiplied by factor *f*(E). This factor was equivalent to the fraction between μ of crossed material at energy E and the μ at 60 keV: μ = μ_voxel_ · *f(E)*. The *f(E)* values were obtained from *ICRU 44 Tissue Substitutes in Radiation Dosimetry and Measurement* [18].

The detector was seen as a set of coplanar points, that belong to a planar surface whose dimensions are equal to those of the radiographic image. Each point simulated the centre of the corresponding pixel of the real detector. A radiation beam from the source was simulated for each one. This distance from the source to the coplanar point (beam path) was divided into *n* equal step increments. The user could set this number *n*. For every *n* the attenuated photon fluence (Φatt_i_) was computed for each energy bin as a product:

$$\Phi att_{i}=\Phi_{i}\prod_{j=1}^{n}e^{-\mu_{j}\Delta x}$$ (1)

where μ_j_ is the linear attenuation coefficient in the middle of the step increment and ∆x is the length of the step (distance between two step increments). An ∆x step length equal to half the voxel size was a good compromise between the computation time and the accuracy of the simulation results.

The dose contribution of each energy bin was calculated with the formula:

$$D_{i}={(\frac{\mu_{en}}{\rho })}_{a}\cdot \psi_{i}\cdot {(\frac{100}{d_{ij}})}^{2}\cdot f_{c}$$ (2)

where (μ_en_/ρ)_a_ is the mass absorption coefficient in air, obtained from *ICRU 44* [18], Ψ_i_ is the attenuated energy fluence of each energy bin (Φ_att i_ · E_i_), (100/d)^2^ is the distance corrective factor and f_c_ is the conversion factor from eV to J. Therefore the total dose (D) incident in the pixel centre is equal to $\sum_{i=1}^{N}D_{i}$ where N is the number of energy bins of the selected beam.

The total dose carried from the source to every digital pixel detector was converted into pixel value (PV) by means of the detector response function. In this application, we created one response function for each simulated detector. The response function was obtained from single response functions according to the photon energy, obtained from experimental measurements. Several direct exposures of the detectors were carried out for the various beam energies and beam intensities for each energy. Two sets of measurements were taken: without added filtration or phantoms (in air) and with a 20 cm PMMA slab phantom positioned near the X-ray tube as an additional filtration. All exposures were carried out without grid in front of the detector and with a source to image detector distance (SID) of 180 cm. A solid state detector (Unfors Xi, Unfors RaySafe, Billdal, Sweden) was placed on the imaging system detector for measuring incident air kerma. The detector was shielded from backscatter radiation by a metal plate inserted into its rear. The mean pixel value and standard deviation of a square central ROI (region of interest) for all acquired images were calculated with ImageJ analyse software [19]. A single response function for each beam energy (PV_X_ = b_X_ + a_X_·ln(K_air_)) for both measurement set-ups was obtained. This kind of response function is common for digital image detectors without specific processing for radiographic projections (such as chest, abdomen, etc.), as also described in IPEM *Report 32* [20].

Since different response functions showed very similar a_x_ values, the b_x_ was subtracted from *PV*_*X*_ value and all the (*PV*_*X*_ *—b*_*X*_ *)* values were interpolated versus air kerma thus obtained for different energies of a single detector. In this way a single factor *a* for all energies was obtained while factor b was calculated as a function of the beam energy for all detectors. We simulated three detectors: two indirect digital radiography (DR), a Philips DigitalDiagnost and a Kodak DR 7500 and a computed radiography (CR) system (Kodak DirectView C.R. 900). The first two implemented a Trixell Pixium 4600 (CsI scintillator) as a detector (pixel size: 0.14 x 0.14 mm^2^); the CR system, instead, is considered of a BaFBr:Eu storage phosphor (pixel size: 0.17 x 0.17 mm^2^). More details about the characteristics of such detectors are available in [21] and [22].

**Fig 1:**
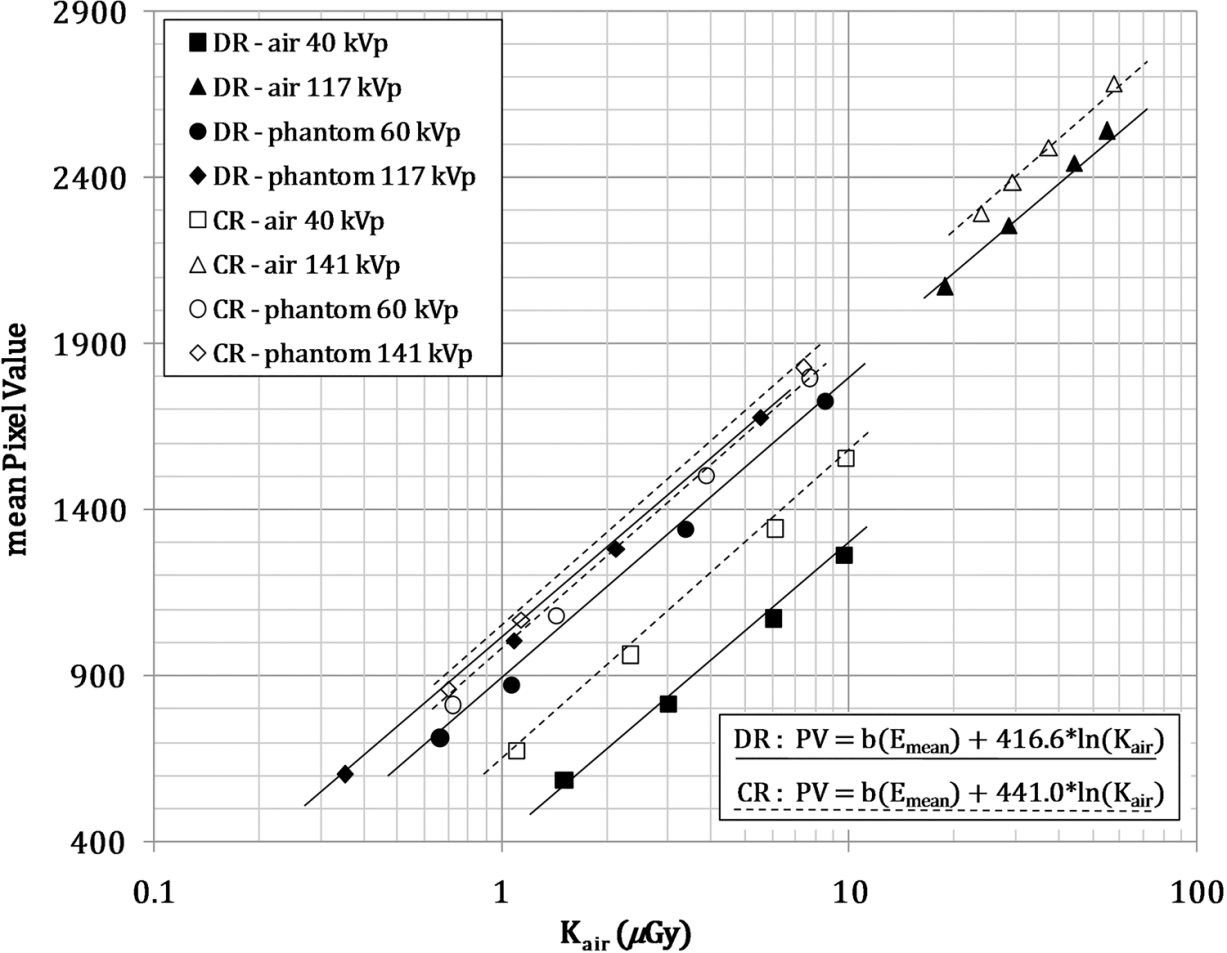
Conversion function from incident air kerma to pixel value for Kodak 7500 DR detector and for Kodak CR 900 detector for four different beam qualities, obtained by combining two different filtrations (air refers to no filtration and phantom to 20 cm of PMMA filtration) with two beam energies. The straight lines represent logarithmic fit functions, continuous lines represent the DR detector and dotted lines represent the CR detector.

Fig 1 shows the in air and in phantom response functions of the lowest and the highest beam energies indicating the extracted unique function for Kodak detectors.

**Fig 2:**
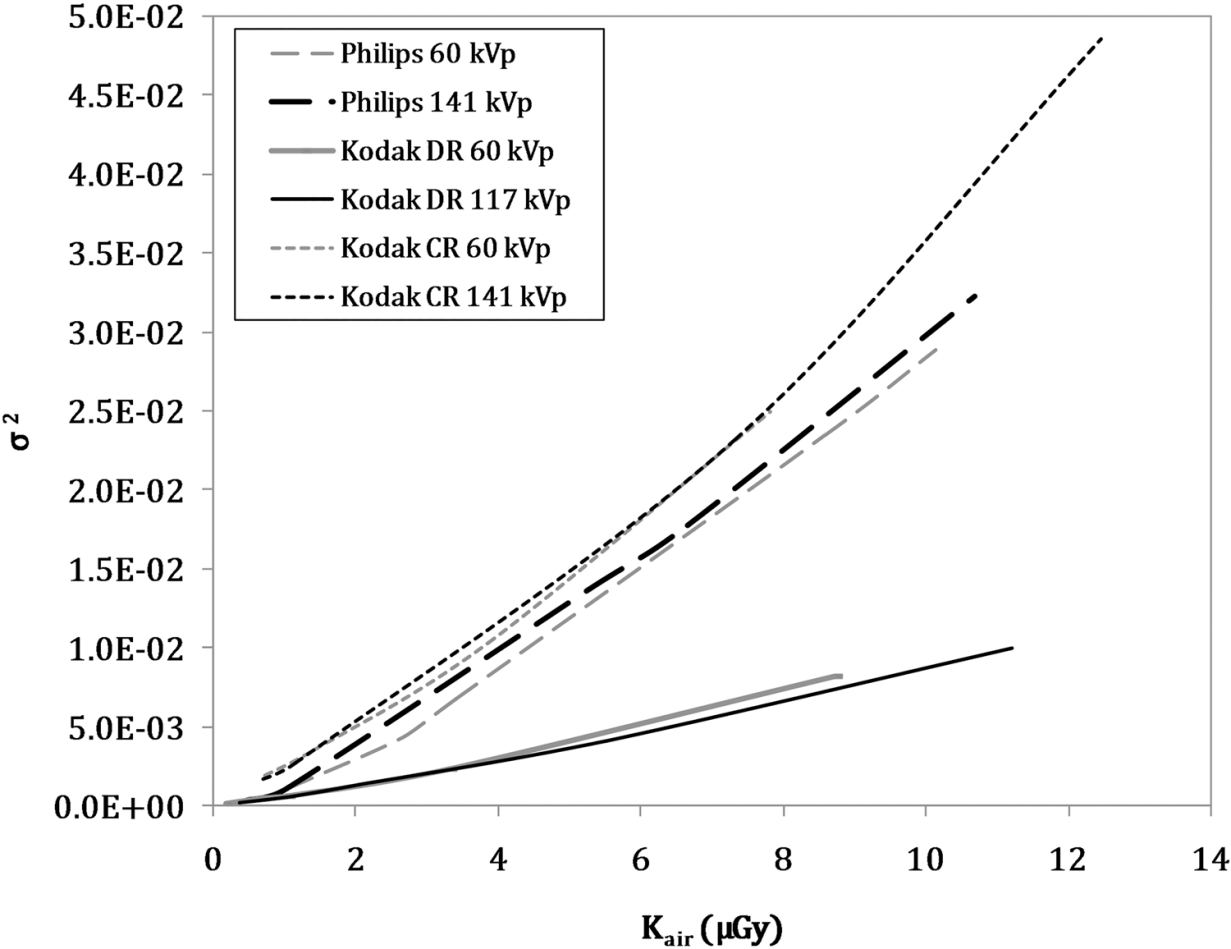
Variance trend of the three detectors versus incident air kerma for two different beam energies. The measurements were taken from a 2 cm x 2 cm ROI square in the centre of the detector.

In addition to the response function, a noise analysis was carried out for each detector. The variance (σ^2^) was decomposed into its basic components [23]: σ^2^ = α·D + β·D^2^ + γ where α is the weight coefficient of the Poisson noise, β that of the multiplicative noise and γ of the additive noise. In Fig 2 the trend of variance measured for the three detectors and two energies is reported.

Considering these variance trends, a dose dependent noise contribute was calculated for each image pixel of the simulated image. In order to obtain Gaussian variables, we used the Box-Muller [24] method: let *U*_*1*_ and *U*_*2*_ be random numbers uniformly distributed over (0,1), therefore *X*_*1*_ *= (-2lnU*_*1*_ *)* ^*1/2*^ *·cos2πU*_*2*_ and *X*_*2*_ *= (-2lnU*_*1*_ *)* ^*1/2*^ *·sin2πU*_*2*_ are a pair of independent random variables with the same normal distribution with mean zero and unit variance. The random numbers (U_1_ and U_2_) for this method were obtained by combining three Tausworth generators (*TausStep*) with a linear congruent generator (*UL*) without mod operation (*LCGStep*) and with different starting seeds (*seed*_*0*_ and *seed*_*1*_) as suggested by S. Mohanty *et al*. [25]. The starting seeds are a result of another linear congruent generator *seed*_*i*_ = *id*_*i*_ *· UL*, where id_*i*_ is a combination of image row and column indices with GPU thread and block numbers since there is no correlated noise. For more details about the random number generation, please refer to [26].

We applied a Gaussian filter to primary beam dose image for the scatter radiation simulation. The Gaussian filter radius (sigma) and the relative coefficient were extracted from a 20 cm PMMA slab phantom experimental image obtained from various beam energies. The phantom was placed on the detector and the radius was evaluated by measuring the penumbra of an edge profile. The sigma depends on the beam energy.

Two grid attenuation factors were extracted: one for the primary beam dose image (f_P_) and one for the scatter radiation dose image (f_S_). For the various beam energies, f_p_ was obtained by means of direct exposures with and without the attenuation grid of the detector and without added filtration or phantom. These images were then converted into doses. We measured the mean pixel value of a central ROI of image with grid and without grid at the same beam energy. The primary beam grid attenuation factor was the ratio between these mean pixel values.

For quantifying the grid attenuation of the scatter radiation, an empirical method was used. We acquired images of a PMMA slab phantom with and without the scattering grid. A comparison of the signal incident to the detector in outside regions of the phantom edges was performed, after subtracting the estimated contribution of the primary beam. The ratio of the scatter contribution with and without the grid provides f_s_ values for different beam energies. In order to validate the method, we simulated the same set of the acquired images and we made a comparison of the achieved scatter results on the actual and the simulated images.

The final dose image was the sum of primary beam dose image and scatter radiation dose image, both corrected for grid attenuation. The final dose image was then converted into gray levels.

In order to compare the simulated images with real digital radiographies, a PMMA slab phantom and an anthropomorphic phantom (3 DIMENSIONAL TORSO, model 602, CIRS, Tissue Simulation and Phantom Technology, Norfolk, Virginia, USA) were used.

Images of five different radiological clinical exams (abdomen, AP lumbar spine, lateral lumbar spine, PA chest and lateral chest) were acquired and simulated with the anthropomorphic phantom. For each exam, five images were acquired with antiscatter grid: one with parameters equal to those used in common practice, two maintaining the same beam intensity and modifying energy mainly to analyse the contrast differences, and the other two maintaining the same energy level but modifying the intensity in order to analyse the noise differences. For each image, some region of interests (ROIs) in homogeneous areas were drawn. The mean pixel and standard deviation values were calculated. Radiopaque markers were positioned on the phantom in order to check the alignment between the real and simulated radiographies and to position the ROIs correctly. Various parameters were studied for each exam: the mean pixel value as a function of beam energy and intensity, standard deviation (SD), signal to noise ratio (SNR) trend in function of the intensity and contrast to noise ratio (CNR) trend in function of beam energy.

**Fig 3:**
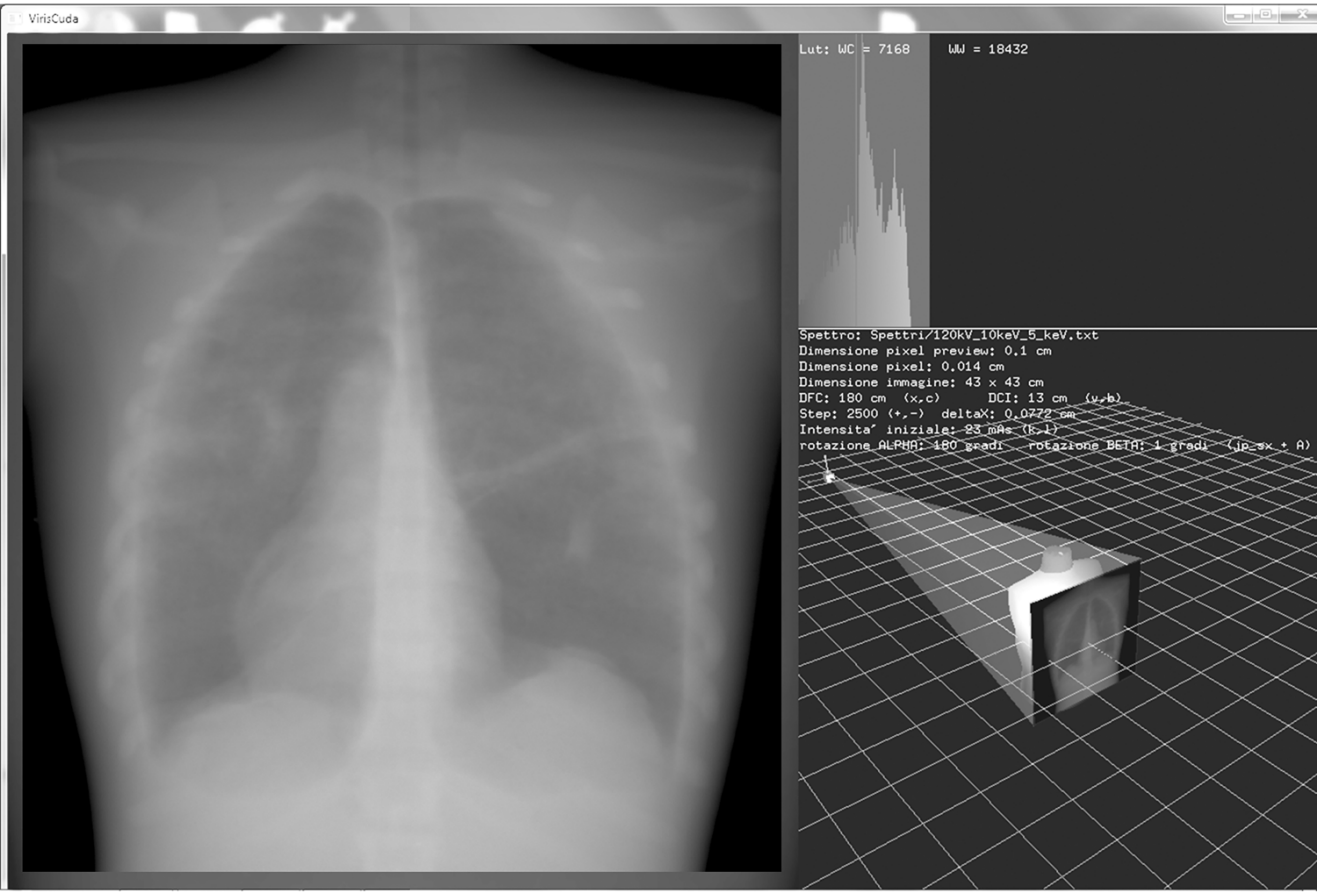
Application display screen: virtual radiological room and simulation parameters in the lower right window; grey value histogram in the upper right window; image preview and radiography on the left hand side.

The application was developed using CUDA technology, GPGPU solution of NVIDIA. A NVIDIA GeForce GTX 680 graphic unit was employed. After the first interface, where the user can select the energy and intensity of the radiation beam, the field dimensions, two angles of source-detector system (i.e, a projection angle that describes the rotation in the transverse plane, and a cranio-caudal angle that specifies the beam tilting with respect to the axial plane), phantom, detector and the linear or sigmoidal look up table (LUT), a user interface display appears (Fig 3). This is divided into three windows: one shows a radiographic image preview, another shows the correspondent gray value histogram, while the virtual radiological room can be seen in the third. The user can modify the beam intensity, step number, the two rotation angles, position source, field dimensions, source-phantom and phantom-detector distance by means of a keyboard and a joystick. A real-time preview update is available at every modification.

The simulation parameters are read by the CPU and then transferred to the GPU which carries out the calculation in parallel of the preview and the final image. A kernel (CUDA function) is performed by a single thread for each pixel of the image.

## Results

**Fig 4:**
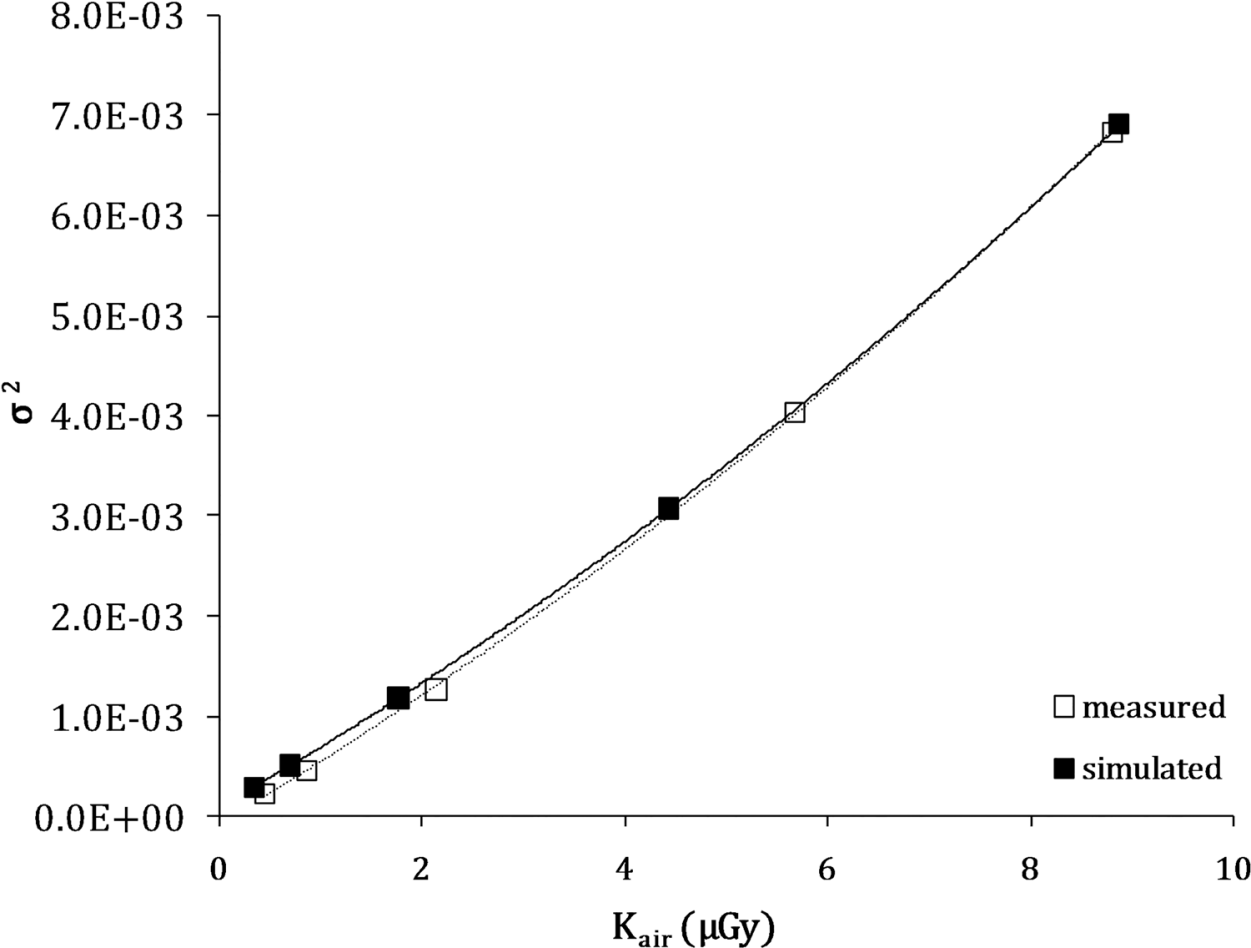
Comparison between simulated and actual variance versus incident air kerma at 102 kVp for Kodak DR 7500.

Several tests were performed in order to analyse the simulated noise and to verify the correspondence with the real noise. Homogeneous PMMA phantom images were considered and a ROI square in the middle. The standard deviation measurements for different detector doses and beam energies were compared. Fig 4 shows an example of noise trend versus air kerma for the Kodak DR 7500 detector and a 102 kVp beam energy. The greatest difference was observed for air kerma values below 1 μGy and was approximately 12%. The other beam energies and detectors showed a similar trend. In order to summarize these results, Table 1 reports the comparison of the noise–air kerma fit coefficients α (poissonian term) and β (multiplicative term). The additive term γ was below 1% of the total noise and not significant therefore it was not included in the table. The maximum difference for fit coefficients was approximately 11%.

**Table 1:** Measured and simulated noise coefficients α and β for the three detectors analysed.

		α			β		
*Detector*	*kV*	*Measured*	*Simulated*	*% diff*.	*Measured*	*Simulated*	*% diff*.
Kodak DR 7500	80	5.803·10^−04^	5.321·10^−04^	-8	2.463·10^−05^	2.215·10^−05^	-10
	120	6.455·10^−04^	5.936·10^−04^	-8	2.253·10^−05^	2.504·10^−05^	11
Philips Digital Diagnost	80	1.666·10^−03^	1.531·10^−03^	-8	4.494·10^−05^	4.770·10^−05^	6
	120	1.808·10^−03^	1.722·10^−03^	-5	6.044·10^−05^	5.853·10^−05^	-3
KODAK C.R. 900	80	1.732·10^−03^	1.536·10^−03^	-11	1.573·10^−04^	1.679·10^−04^	7
	120	2.328·10^−03^	2.084·10^−03^	-11	1.376·10^−04^	1.475·10^−04^	-7

**Fig 5:**
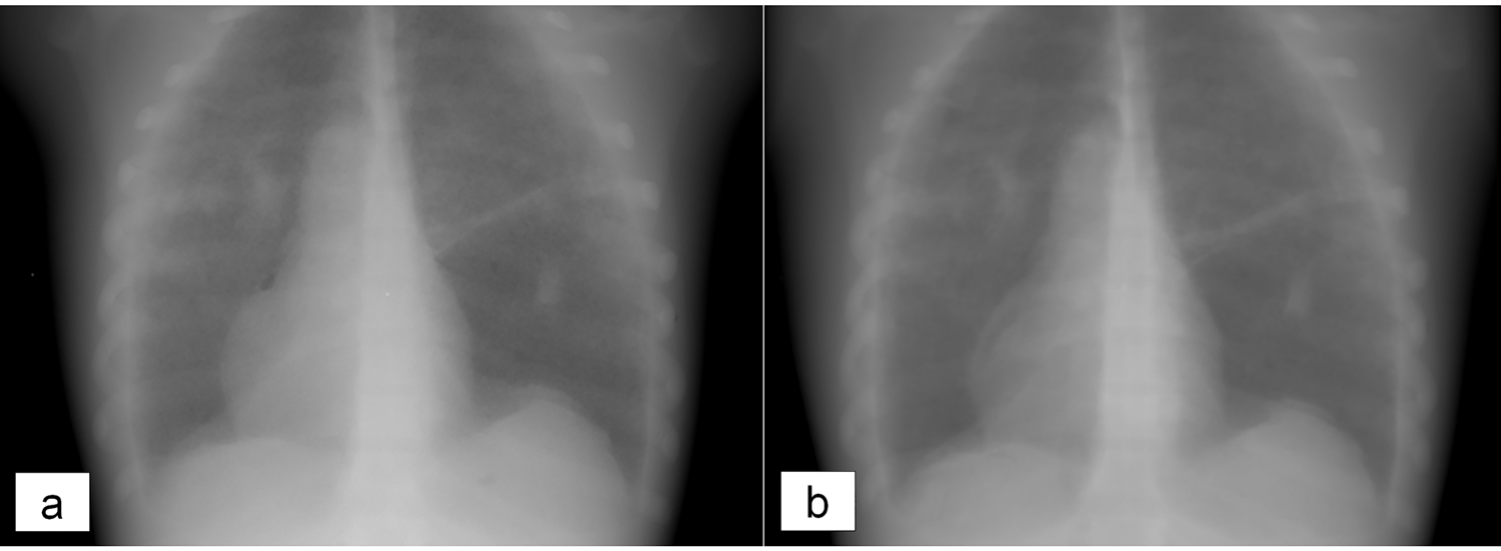
Comparison between a simulated (5a) and a real (5b) PA chest radiography of anthropomorphic CIRS phantom. The real image was obtained with a Philips Digital Diagnost with 117 kVp and 2 mAs. Both the images are raw images.

Fig 5 shows an example of a comparison between a simulated (5a) and a real (5b) radiography of the anthropomorphic phantom. The average pixel values and standard deviations obtained for ROI positioned in images of AP and LL chest, AP and LL lumbar spine and abdomen for both real and simulated radiographies are shown in Table 2. In terms of average pixel values, the maximum differences for digital direct detectors were below 10%, while a maximum difference of 15% was observed for computed radiography system. Standard deviation differences were in accordance with a maximum difference of 20%. This difference concerns the order of noise homogeneity required overall the area of the detectors in our routinary performed quality assurance tests.

**Table 2:** Mean pixel value and SD for several ROIs of five examinations carried out with common exposure parameters, for the three detectors analysed.

		Philips DigitalDiagnost				Kodak DR 7500				Kodak C.R. 900			
		mean PV		SD		mean PV		SD		mean PV		SD	
Examination	ROI position	MEAS	SIM	MEAS	SIM	MEAS	SIM	MEAS	SIM	MEAS	SIM	MEAS	SIM
ABDOMEN	SOFT TISSUE	14780	14986	94	78	1141	1201	11	11	1070	1091	22	23
PA CHEST	LUNG	12413	11915	90	95	1607	1699	10	11	1005	1090	27	23
	MEDIASTINUM	14416	14477	167	187	1292	1342	14	16	713	733	38	33
LATERAL CHEST	LUNG	11666	11215	91	86	1734	1807	14	13	1560	1636	25	21
AP LUMBAR SPINE	SOFT TISSUE	11665	11999	82	97	1502	1595	13	15	919	1040	26	21
	VERTEBRAL	12354	13047	60	48	1372	1423	10	10	733	845	31	25
LL LUMBAR SPINE	VERTEBRAL	14054	14628	114	101	1291	1170	21	20	1014	980	29	24

**Fig 6:**
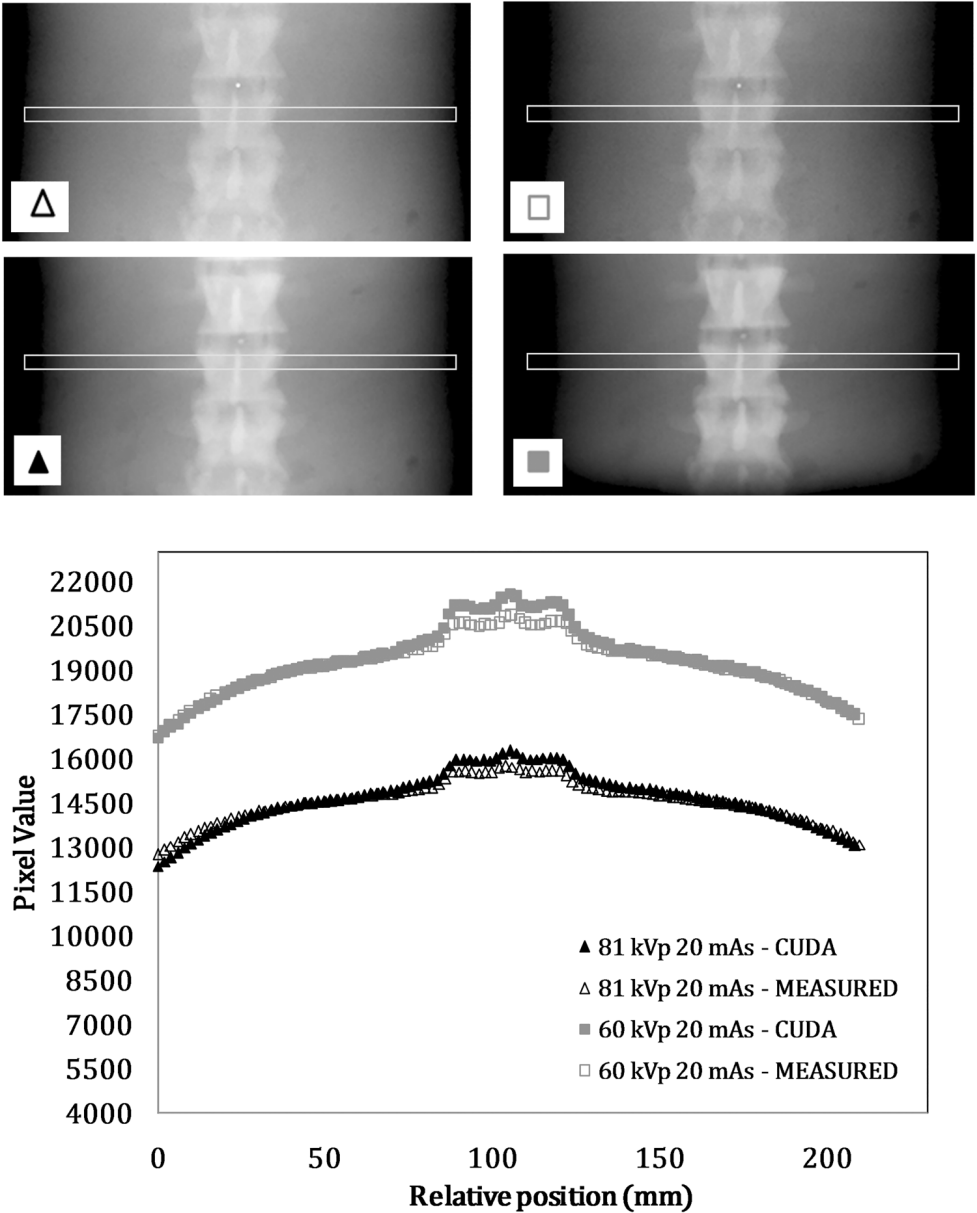
Qualitative comparison between measured and simulated images for Philips DigitalDiagnost: particular of two lumbar spine images with different exposure parameters. See below the acquired images and simulated images, pixel value profiles obtained from a rectangular ROI.

**Fig 7:**
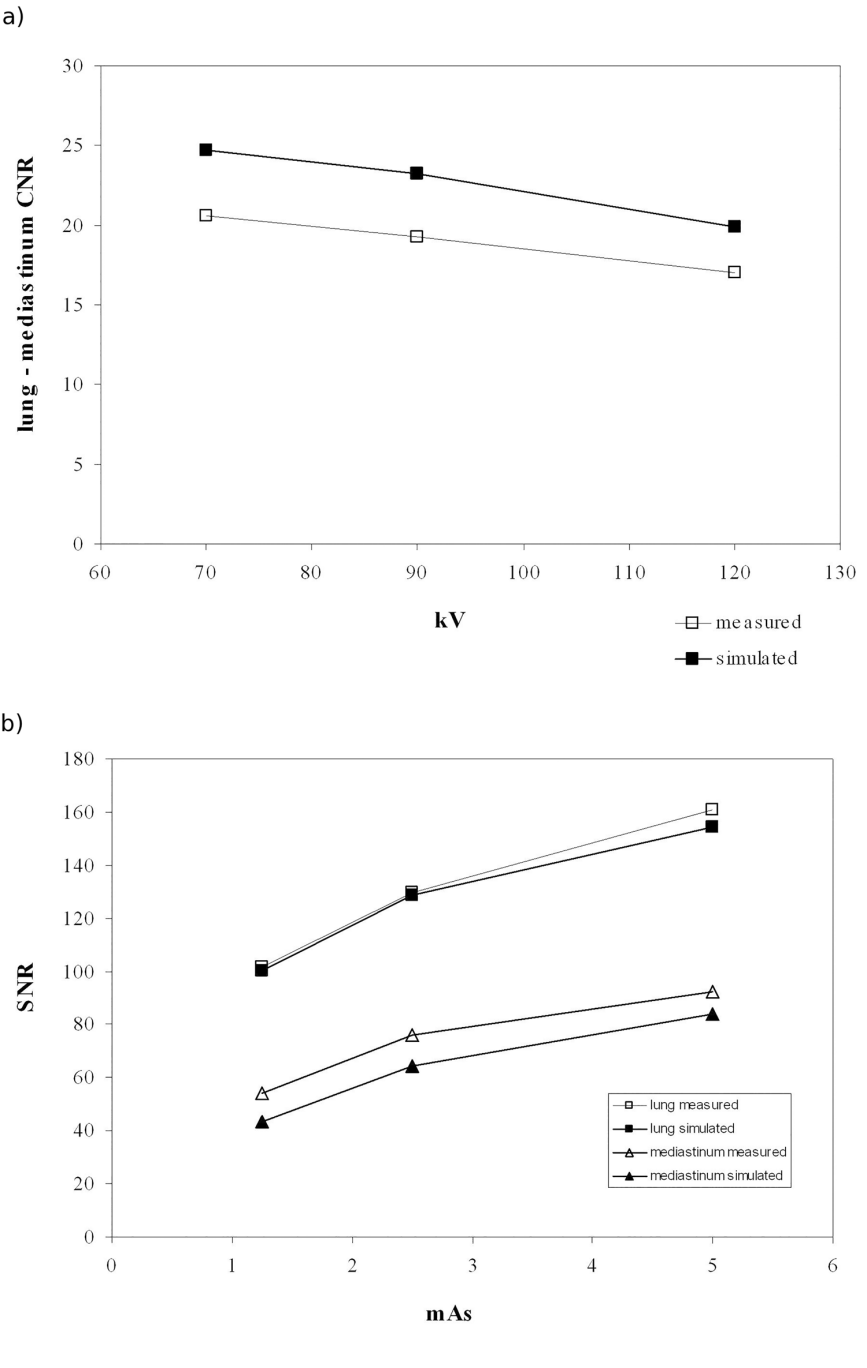
(a) Contrast noise ratio between two ROIs positioned in the phantom mediastinum and lung tissues of a PA chest radiography for real radiographies (measured) and simulated radiographies, for three different kVp values. (b) Signal noise ratios for three different mAs values of the same ROI position.

Another qualitative comparison between simulated and acquired abdomen images concerning a lumbar spine image with two different beam energies is reported in Fig 6. A reduction in voltage causes an increase in contrast in the simulated image just like the real image. The relative noise trend versus air kerma and contrast noise ratio between two phantom tissues versus the beam energy were analysed for the various examinations. For example Fig 7(A) shows the trend of the contrast noise ratio evaluated between lung and mediastinum tissues versus the beam energy for a chest PA projection. Differences between real and simulated data were below 25%, and the relative decrease of contrast noise ratio ranging from 70 to 120 kV was -17% for real data and -19% for simulated data. In Fig 7(B) the signal to noise ratio for the same projection and tissues versus the tube current time product were shown, with a maximum difference of 20% between real and simulated data.

Lastly, the calculation time with anthropomorphic phantom images was determined. The calculation time depended on the beam energy, matrix size and number of steps. For different radiographic projections, GPU calculation time ranged from 0.8 to 2.3 seconds. It includes also the data transfer time between host and device. The same simulations were performed without GPU with a usual CPU (PC Intel Core i7 3770 3.4 GHz, 16 GB RAM) in order to make a comparison which resulted in a 200 times greater calculation time as reported in Table 3 for Kodak DR 7500. The outcome was similar for the other detectors.

**Table 3:** Comparison of calculation time between GPU and CPU for various clinical exams with anthropomorphic phantoms for the Kodak DR 7500 detector (pixel size 0.14x0.14 mm^2^).

EXAM	GPU (s)	CPU (s)	CPU (s) / GPU (s)
Abdomen	1.22	257.2	210.1
AP vertebral column	0.98	215.1	219.2
LL vertebral column	1.29	215.0	167.0
PA chest	1.86	292.4	156.9
LL chest	2.06	270.0	131.0

## Discussion and Conclusions

A digital X-ray imaging system simulation tool based on graphics processing unit (GPU) was presented. This aim of the study is to develop a realistic and efficient tool for training operators and dose optimisation studies. The “realistic” characteristic was evaluated by the comparison between simulated and real images, while efficiency was mainly related to the calculation time and the quality of the user interface.

Table 4 shows a comparison between this study and other papers cited in literature with the aim of simulating X-ray digital radiography systems. Most of them used CPU platforms, except for the Jia works [10] who simulated a CBCT system, bearing in mind the single planar radiographies required for reconstructing the tomographic image. Among these studies, a validation with a SNR comparison between real and calculated images was performed only for Moore’s simulation system [7], while no or only partial validation (profile comparison) was carried out for the other studies.

**Table 4:** Comparison of the simulation elements and the results obtained in this study and from literature on similar subject. “n.d.” means not declared.

Reference	Validation by comparison with actual images	Virtual phantom	Noise	Radiation beam spectrum	Number of digital detectors considered	Pixel size (Image matrix)	Calculation time	Focal spot size	Scatter contribution	Scatter removal grid
[1] Duvachelle2000	No	CAD model	Poissonian	Polyenergetic	1 (virtual)	0.2 mm (500 x 500)	from 0.1 s to several hours	Actual dimensions by means of adjacent points	No	No
[2] Lazos 2003	Profiles and Scatter to Primary ratio comparison	geometric or voxel	Specific noise issues not investigated	Polyenergetic	1	0.7 mm (180 x 180)	4 hours	Punctual	Yes	Yes
[3] Fanti 2005	No	voxel	Not investigated	Polyenergetic	1 (Film)	0.25–0.5 mm	n.d.	Punctual	No	No
[5] Winslow 2005	No	voxel (human project)	Poissonian	Single energy	1 (virtual)	n.d.	6 weeks for scatter	Punctual	Yes, precalculatedby Monte Carlo	Yes
[7] Moore 2011	SNR	voxel rando	Locally normalized to incident dose, frequency dependent	Polyenergetic	1 CR	0.8x0.3 mm^2^ (700 x 1000)	45–90 minutes	Punctual	Yes, empirical with Rando measurements	No
[10] Jia 2012	Profiles and noise comparison (tomographic images)	voxel	Poissonian	Polyenergetic	1 (Cone Beam flat panel)	0.78×0.78 mm^2^ (512 × 384)	2 s for analytical and 7 hours for Monte Carlo scatter	Punctual	Yes, Monte Carlo	No
This study	CNR, SNR, profile comparison	voxel	Poissonian. Multiplicative and Additive	Polyenergetic	3 (2 DR and 1 CR)	0.14x0.14 mm^2^ (2500 × 3070)	<3 s	Punctual	Yes, empirical based on primary beam data with gaussian filter	Yes

Dose reduction in digital radiography is limited by the progressive increase in noise and noise proves to be incompatible with a correct diagnostic information below a defined threshold. Consequently, in order to obtain valuable suggestions for optimisation strategies, the simulation tool should account for the real noise resulting from a dose reduction correctly. Unlike other studies, three noise contributions (poissonian noise, multiplicative and additive) were considered and simulated. A good equilibrium was found between simulated and measured poissonian and multiplicative noises.

**Fig 8:**
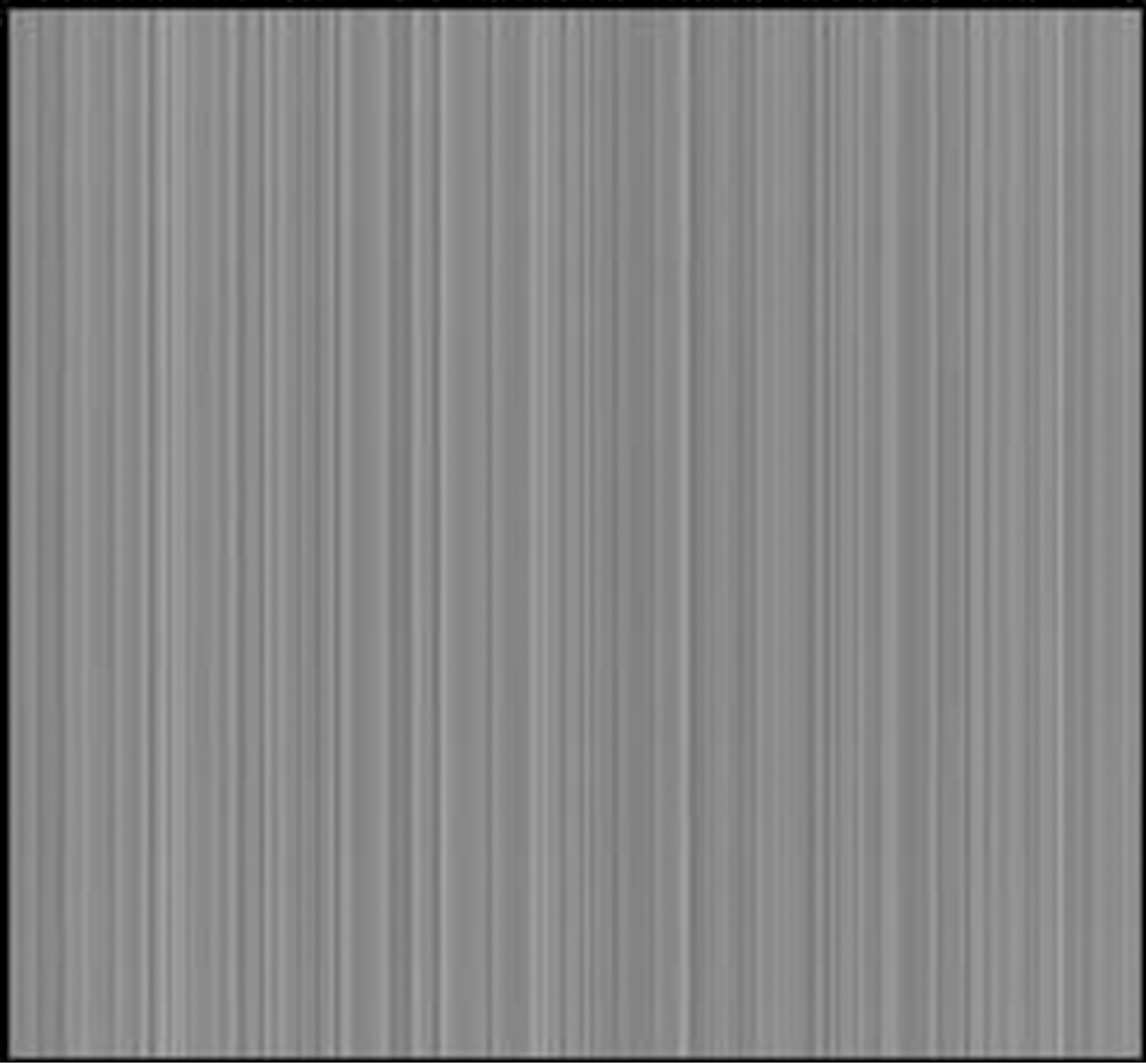
Example of a 20 mm PMMA phantom simulated image obtained with a wrong choice of index for random number generation equal to: id_1_ = blockIdx.x · blockDim.x + threadIdx.x and id_1_ = blockIdx.y · blockDim.y + threadIdx.y Streak artefacts are associated with correlated noise.

The additive contribute to total noise was not relevant or significant. A good equilibrium was also obtained between measured and simulated noise variance versus incident air kerma for all detectors and for various X-ray energies. This result was possible thanks to the random generation approach previously described, which combines several Tausworthe generators [27], linear congruential generators and Park-Miller algorithm [28] in order to improve noise generation. It is important to note that the choice of the initial seed (*seed*_*i*_ and *id*_*i*_) is essential: correlative *id*_*i*_ can lead to correlative noise. Fig 8 shows an example of what occurs when one chooses the wrong initial seed which leads to visible “strips of noise” as a consequence of correlative noise.

Variations of the beam energy (kVp) are often used in radiodiagnostic practice to account for anatomical differences or different diagnostic issues. The influence of the beam spectrum was simulated considering the energy distribution of the photon fluence divided into 5 keV bins and the various relative attenuation coefficients across the virtual phantom. The virtual body consisted in a 3D voxel matrix generated from CT images. From the HU voxel value, a linear attenuation coefficient was obtained which was corrected for the beam energy and density of the crossed tissue. This energy correction was introduced in order to account for the real trend of radiation attenuation considering the spectrum variation across the volume. The simulation of the various exams with anthropomorphic phantom showed that the trend of standard deviation versus kVp, signal to noise ratio versus mAs and contrast to noise ratio versus kV were in agreement with those measured. Only one anthropomorphic phantom was used. The next step was to include an anonymized real patient dataset in the software in order to evaluate the differences caused by patient dissimilarity and the diseases diagnosed from a traditional X-ray examination.

The commissioning of a new detector is another issue that requires care when training or choosing a parameter. In this case, it may be useful to compare this technique with another detector. Three detectors were implemented in this study: two DR (Philips DigitalDiagnost and Kodak DR 7500) and one C.R. (Kodak DirectView C.R. 900). Each detector has a proper response function, noise and specific features depending on the manufacturer. To our knowledge, different detectors have not yet been analysed in other studies in literature on simulation tools. The two digital direct detectors were both Trixell Pixium 4600 (CsI scintillator) but they are characterised by different response functions and, more importantly, different noise. The graph in Fig 2 shows that, for the same incident dose, the Philips Digital Diagnost has a greater variance than the Kodak Direct 7500. The noise ratios obtained for clinical examinations varied greatly for the three detectors, since the Kodak DR 7500 values were approximately 3 to 5 times greater than the Kodak CR 900 images. In this context, the differences between real and simulated signal to noise ratios of 20% are not significant. They are compatible with useful considerations concerning the various ways of using the detectors and the different exposure parameters necessary for obtaining the minimum image quality requirements.

A comparison regarding GPU and CPU calculation time was carried out. The advantage of using the graphic processing unit as a CPU co-processor is evident since it allows for real-time interactivity between the user and software without jeopardising reliability and precision.

A comparison with recent studies in literature highlights similar results and calculation times of tenths of minutes for CPU based tools [11]. Only a few seconds are required for calculating GPU based simulations of the primary beam, while the Monte Carlo addition of scatter contribution could take several hours even with recent hardware platforms [10]. It is not easy to compare the calculation times of this study and other studies published due to the different matrix size and simulation parameters used, yet when the goal is a projection radiography it is not essential to consider the differences in fractions of a second.

A limitation of this study was that a virtual punctual source was used instead of a geometrical focal spot with real dimensions. As shown in Table 4, this was only done by [4] summing the number of the beams generated by several single point sources arranged next to one another in a small matrix of dimensions similar to those of the focal spot. This enabled us to highlight the differences of spatial resolution related to the focal spot in the simulation of virtual phantoms generated as CAD (Computer-Aided Drafting) models. In this study a voxel phantom with 0.5 mm voxels was used, while the dimensions of the focal spots used in traditional X-ray tubes were approximately 0.6 and 1.2 mm. During quality control tests, an RMI Focal Spot Test with a bar pattern situated 15 cm from the detector surface and 46 cm from the source was carried out. It is possible to distinguish a 3.8 lp/mm pattern with a 0.6 mm focal spot, while a 2.3 lp/mm pattern can be seen with a larger focal spot. These spatial frequencies are evidently greater than that one of the adopted virtual phantom (1.6 lp/mm) and therefore the differences between large and small focal spots would not be highlighted. It is also important to consider that the choice of the focal spot is not a fundamental parameter for training or optimisation purposes, as using small focal spots for bone examinations and large focal spots for abdomen or chest examinations are standard procedures. For these reasons the implementation of a real focal spot, with related consequences also in terms of calculation time, was not considered a priority in this study. Higher resolution paediatric phantoms and finite size focal spot implementation could be considered for future developments, since the use of small focal spot is particularly relevant in the pediatric field.

Another limitation of this study was the empirical and approximate method used for simulating the scatter component of the image. The approach used is similar to that adopted in [7], which requires an empirical model based on scatter fraction measurements for a single anthropomorphic phantom and adaptation for other virtual phantoms based on patient data. Measurements with a geometrical phantoms were taken in order to consider the scatter component of the signal and its profile. Other methods such as Monte Carlo [5,10] are more accurate, especially in the bone region, but a long calculation time is required which does not allow for real-time interactivity with the application by the user.

Most of the studies published do not carry out the grid attenuation of primary and scatter radiation, which is an essential element always used in the digital conventional radiography of adults. The scatter to primary fraction can reach values ranging between 0.7 to 0.9 for a chest radiography [29], but following the grid attenuation this fraction is generally reduced below 0.3 [30]. The primary and the scatter attenuation factors of the grid for several beam energies were evaluated and implemented in the final image calculation. Although the scatter component of the image is empirically simulated and does not coincide with the actual scatter distribution, the presence and the simulation of the grid effects limited the associated errors. In the balance between tool velocity and accuracy, efforts were concentrated towards the first objective, and the results demonstrate that this enables us to carry out a valid simulation and obtain useful information. Future research will be focused on the implementation of a GPU Monte Carlo method for improving the accuracy of this contribution.

The software does not provide an estimation of the radiation dose, but after selecting an anatomical district and a patient type, the dose indicators’ variations dependent on the changes of kV and mAs can be easily estimated based on the theoretical relationships. However, dose estimation tools are planned for future developments, in a first step as dose area product indication (i.e., the quantity usually provided by digital X-ray systems) and in a second step as organ dose estimation. As shown in Table 4, only few previous studies made a comparison between the actual and simulated images for the validation of the simulation results. All of them consider physical measurements, such as SNR, and the comparison of intensity profiles. The investigation of the perceived image quality could be a second level of validation, implemented by selecting a proper set of phantoms with the possibility to rate the scores of the image quality parameters, and provide for an evaluation by experts in the field. To date we receive preliminary and encouraging comments about the quality of the simulated images by expert radiologists. For future work we would like to consider an in-depth investigation of the perceived quality of simulated images.

In conclusion, we can affirm that this application proved to be efficient and realistic. After testing the software with other anthropomorphic phantoms of various sizes, it would be possible to use it for training operators and carrying out dose optimisation procedures in a radiological department, thus saving temporal and human resources and radiological room occupancy time.
